# Bidirectional propagation of tilting domain walls in perpendicularly magnetized T shaped structure with the interfacial Dzyaloshinskii-Moriya interaction

**DOI:** 10.1038/s41598-018-36523-9

**Published:** 2018-12-21

**Authors:** Jaesuk Kwon, Hee-Kyeong Hwang, Jung-Il Hong, Chun-Yeol You

**Affiliations:** Department of Emerging Materials Science, DGIST, Daegu, 42988 South Korea

## Abstract

Understanding of domain wall (DW) propagation in a complex structure is an essential first step toward the development of any magnetic-domain based devices including spin-based logic or magnetic memristors. Interfacial Dzyaloshinskii-Moriya interaction (iDMI) in the structure with broken inversion symmetry induces an asymmetrical DW configuration with respect to the direction of in-plane field. Dynamic behaviors of field-driven DW within the film with perpendicular magnetic anisotropy is influenced by DW tilt from the iDMI effect and the corners in the T-shaped structure of the DW path. Images from Kerr microscopy reveal that the iDMI effective field contributes to a tilted structure of DW configuration and evolution along its propagation. With the combination of iDMI and T-shaped structure, we observed two distinguished bidirectional DW propagations in two output branches and distinct arriving times at the destination pads with a uniform external field. Micromagnetic simulation results is compared with the observed dynamics of a DW configuration in the structure providing an additional confirmation of the interpreted results.

## Introduction

Efficient driving of magnetic domain wall (DW) in a complex structure is receiving growing attentions as it is an essential prerequisite for the development of spin-based logic or memristor devices, whose operations are based on the domain reversals and/or transportations^1–3^. In the applications of ultra-thin ferromagnetic films, Pt under- and/or over-layers in contact with neighboring Co layer as in the Pt/Co/Pt and Pt/Co/Ta stacks are attributed to induce the perpendicular magnetic anisotropy (PMA)^4–8^. Another unique feature called the interfacial Dzyaloshinskii-Moriya interaction (iDMI) originated from strong spin-orbit coupling in adjacent heavy metal layer is also induced in association with asymmetric layer structure around the interface. An inversion symmetry of stack is broken by introducing either different materials or different thickness of the layers around the ferromagnetic layer^9–12^. The other symmetry breaking is studied for Pt and Ta as under and capping layer due to their relatively large difference of spin Hall angle (SHA), 0.07 and −0.12, which is important in current-driven DW motion^13–15^.

The role of iDMI effect for the spintronics application, changes in the velocity of current-driven chiral tilting DW along a curved PMA nanowire has been reported by C. Garg *et al*.^16^. Recent studies for the DW based devices have primarily been focused on 1-dimensional wire systems, but a practical device fabrication requires an expansion to complicated 2 or 3-dimensional structures consisting of numerous 1-D straight or bent wires as well as the connections of them. Complex structure including multi-branches would unavoidably accompany the novel phenomena associated with structural defects at all bifurcation points (T- and/or Y-shaped structure). It is known that the presence of iDMI leads to different DW depinning fields for structural defects such as notch, depending on the relative orientations of the spins inside the DW with respect to the defect geometry^17–20^. It has been reported that iDMI stabilized Néel DW type and the Walker breakdown is suppressed to higher external fields due to the effect of iDMI^21^. The iDMI introduces an evolution of Néel DW spin configuration, wherein a Bloch DW configuration would be favored in the absence of iDMI^22,23^. The iDMI is known to cause the tilting of the magnetization direction inside the DW as demonstrated by Je *et al*. with their Kerr microscopy imaging technique^24,25^. The DW motion driven by either current or field is affected by iDMI effect and accordingly the creeping or flowing DW velocities depend on the magnitude of iDMI^26,27^. The iDMI effectively induces an internal field, H_DMI_, setting a preferred DW chirality as the magnetization direction progressively changes across the DW from up to down or down to up domains^28,29^. Furthermore, iDMI effect possibly induces an asymmetric domain expansion speed with DW moving with asymmetric in-plane field speeds depending on the direction of effective fields, H_DMI_. Therefore, the chiral DW motion with iDMI effect in the wire structure requires the detailed consideration of DW spin structures.

In the present report, we present the dynamic evolution of DW configuration within a 2-dimensional T-shaped microwire junction structure with the consideration of iDMI effect. With the combined effects of the iDMI and the corner structure in the T-shaped junction, a DW proceeds along the wire structure with a tilt angle corresponding to the chirality of DW with respect to its propagation direction. As the DW turns around the corners associated with T-junction, asymmetric tilted DW bifurcates and turns around the two corners with two different cornering speeds. As a result, we obtained distinguished bidirectional DW motions for a given uniform external field. The dependence of DW configuration and location in a T-shaped structure has been investigated with direct observations of DWs configuration and position by utilizing Kerr microscopy technique.

## Results and Discussion

### Film characterization

**Figure 1:**
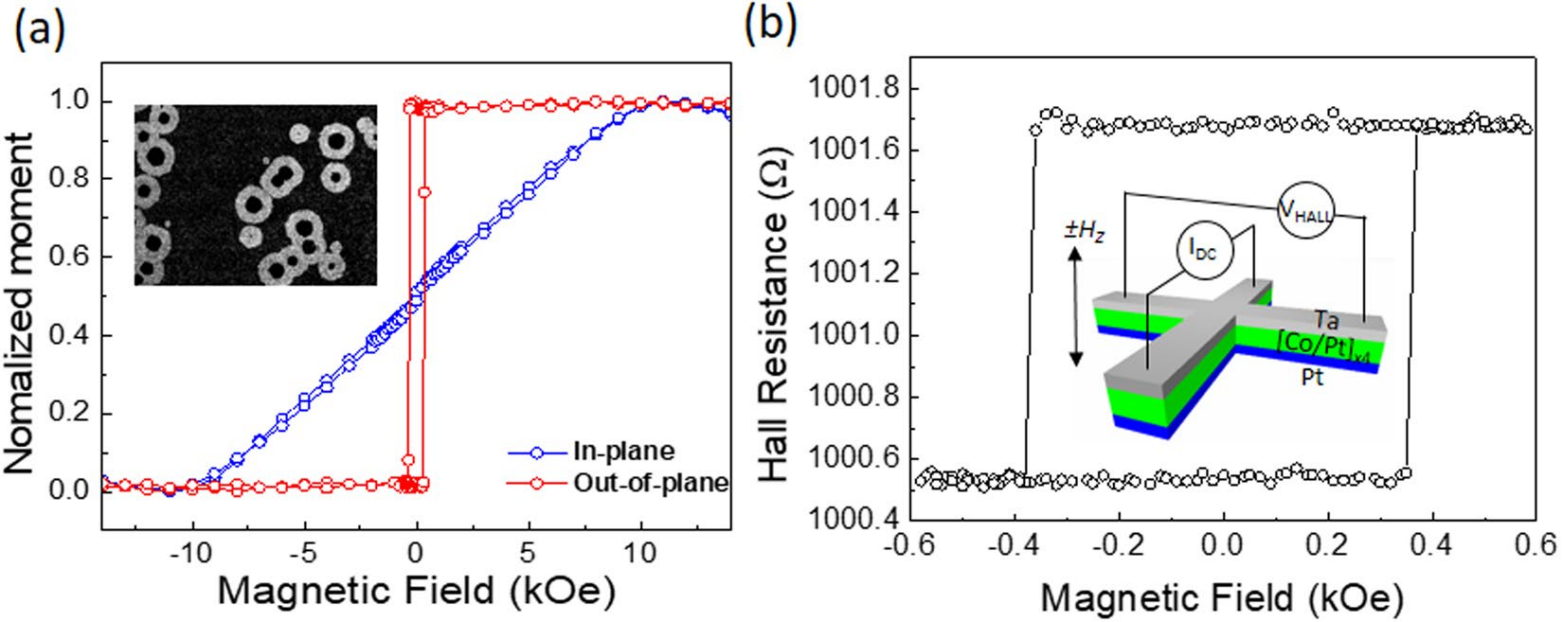
(**a**) Normalized VSM signal along the applied out-of-plane and in-plane field for the film. The inset image is overlapping of the captured two images for symmetric expansion of circular magnetic domains in the film by perpendicular field. The expansion corresponds to bright (dark) contrast with up (down) domain. (**b**) Hall resistance changes as function of applied field with a dc current of 100 μA in the patterned Hall cross structure. The inset depicts a schematic illustration of a Hall cross structure and measurement setup.

Ta (5 nm)/[Co (0.3 nm)/Pt (0.3 nm)]_×4_/Co (0.5 nm) with a Pt (4 nm) capping layer was prepared on SiO_2_ covered Si substrates employing magnetron sputtering deposition technique. The spin-orbit related phenomena can be induced due to the symmetry breaking at the interfaces of heavy metal/ferromagnetic metal/oxide layers, where strong spin-orbit coupling is expected in the heavy metal layer^13^. Additionally, a multilayered film comprised of the FM layer sandwiched between HM layers can enhance the iDMI effect^30–32^. Magnetic characterizations with VSM measurement indicates that the film exhibits a PMA, with a saturation magnetization of *M*_*s*_ ≈ 1013 kA/m and effective anisotropy energy, *K*_*eff*_ ≈ 0.51 MJ/m^3^, as shown in Fig. 1(a). To validate the PMA magnetically and electrically, the anomalous Hall effect (AHE) has also been measured with a patterned Hall bar device^33,34^.

The Hall resistance switching at the coercive field, Δ*R*_*Hall*_ ≈ ±1.2Ω, corresponding to the switching between up or down magnetization in Hall bar area, is obtained as shown in Fig. 1(b). AHE hysteresis loop shows the coercive field, *H*_*c*_ ≈ 360 Oe. The detection of Δ*R*_*Hall*_ implies that a DW propagates through the Hall bar area or a domain may be nucleated and is moving cross the Hall bar.

### Device structure and Kerr images of magnetic domains and domain walls

**Figure 2:**
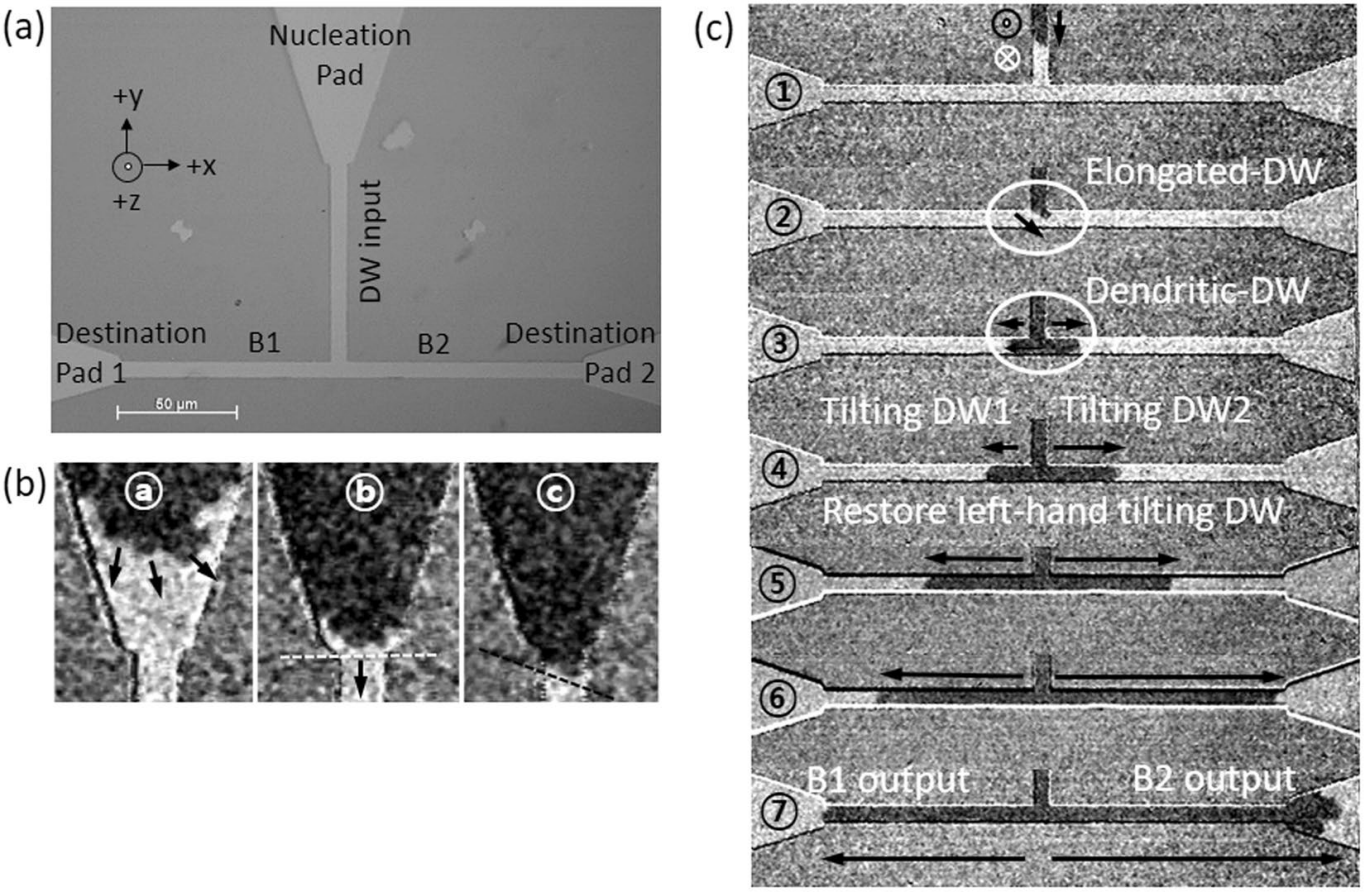
(**a**) Microscopy image of T-shaped device comprised of nucleation pad, DW input wire, two outputs (B1, B2), and destination pads at the end of branches. (**b**) The initialized process of DW in the input wire. ⓐ Irregular type of DW expansion in nucleation pad. ⓑ DW approaches towards conjunction (white dash line) between the nucleation pad and wire. ⓒ The handedness of DW surface rotation is decided once it pinned at the conjunction between the nucleation pad and the input wire. (**c**) Kerr imaging of field pulse-driven DW creep motion in the structure. ①–② An up-down DW passes via the input wire. ②–⑥ show that the DWs propagate into the output branches. At the junction, a DW elongates towards favorite output branch, B2 first. ⑦ The DW2 approaches the destination pad 2, which is attached to the B2. The DWs in the white circle indicates an evolution of DW configuration at the junction.

In order to precisely drive the DW motion in the micropattern-structure, the magnetic field pulses were applied as magnetic domain propagation was recorded using Kerr microscopy to track the field-driven DW dynamics along the T-shaped structure. Figure 2(a) shows a T-shaped structure consisting of interconnected input wire, 5 μm wide and 80 μm long, with the domain nucleation pad at the top.

For the initialization of DW position in the structure, the sample was initially saturated with an external field of ∼−1kOe along the -z-axis, which is greater than the coercive field determined from AHE measurement with the Hall bar structure. The saturation magnetization can be confirmed from the Kerr microscopy image of entire structure with a uniform bright contrast. By applying an opposite field pulse along the + z-axis with a pulse duration time of 500 ms and the magnitude of *H*_*pulse*_ ≈ +280 Oe below the coercive field (~360 Oe), nucleation of several circular domains was initiated in the nucleation pad. One of the domain was then driven to enter the input wire, as shown in Fig. 2(b). DW motions are captured repeatedly with Kerr microscope as perpendicular field pulses are applied as shown in Fig. 2(c).

Successive applications of the short field pulses with time durations of less than 100 ms have been used to drive the DW through the input wire with a controlled velocity of around 100 μm/s. From the Kerr images, a left-handed tilt of up-down DW with respect to its propagation direction in the input wire can be readily recognized in Fig. 2(b,c), reflecting the influence of iDMI. The motion of the left-handed tilting up-down DW is always accompanied by a clock-wise DW surface rotation with respect to the right edge of the input wire. Initial formation of the tilt for the DW in the input line can be induced at a linkage point between the large nucleation pad and the narrow input line, where the line DW can be recognized to be tilt unevenly as shown in Fig. 2(b,c) black dash line. The formation of tilted state would be stabilized by the presence of iDMI effective field during the propagation through the input line in Fig. 2(c-①). As the DW reaches the T-junction, the distinct motions of the tilted DW at the two corners for the left and right turns need to be considered.

**Figure 3:**
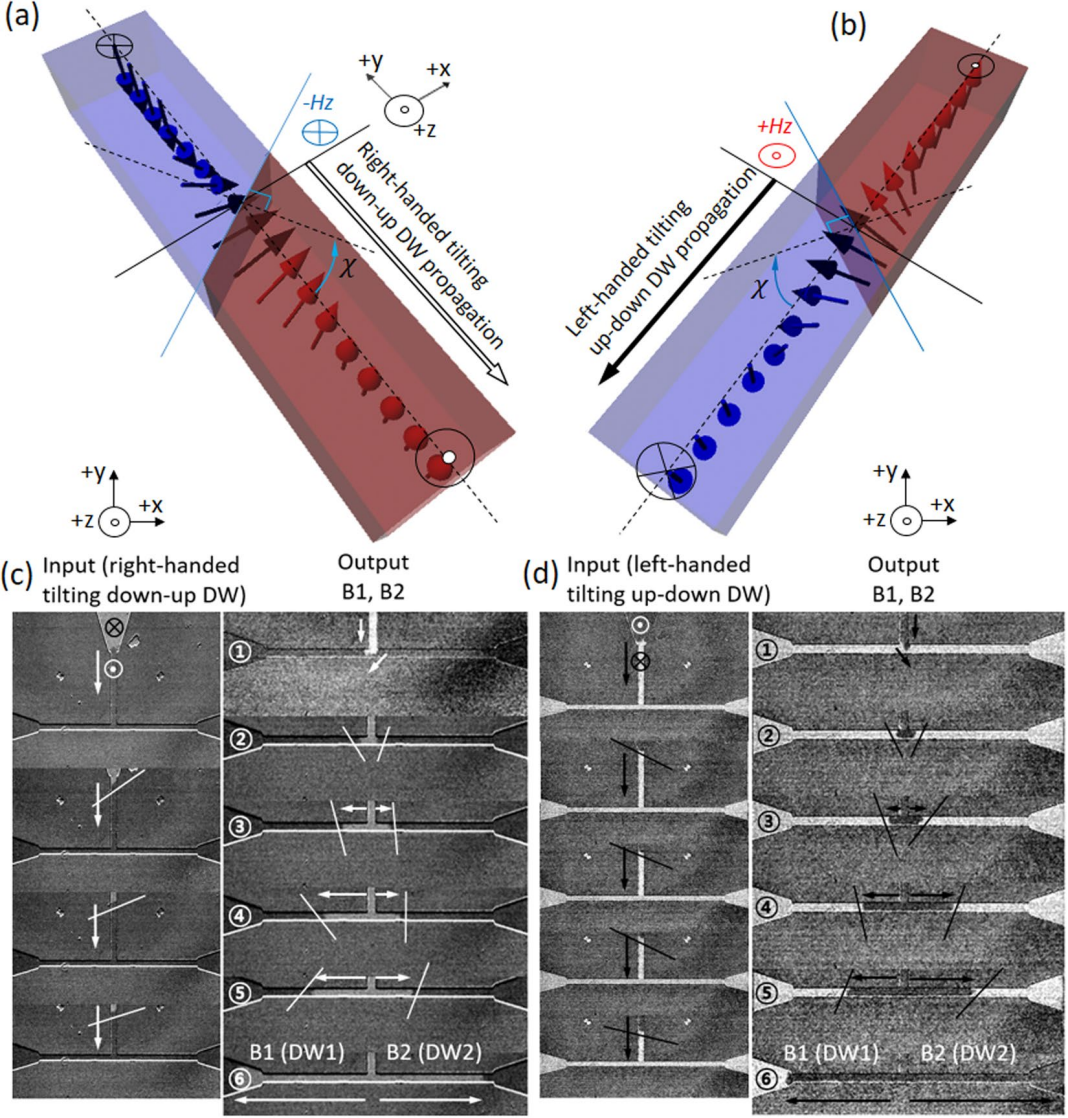
(**a**) Illustration of right-handed tilting down-up DW, (**b**) left-handed tilting up-down DW in input wire. Differential Kerr microscopy images for DW dynamics as a DW flows from input wire by continuous field. (**c**) Right -handed tilting down-up DW propagates via input wire, junction, and branches. ①–⑥ DW tilt angle has been oriented due to the propagation directions from bifurcated junction to two outputs. (**d**) Left-handed tilting up-down DW moves via the T-structure. The dendrite-DW configuration has been observed in both up-down and down-up DW at the bifurcated junction.

As can be seen in Fig. 2(c-②), the DW exhibits an asymmetrical expansion with elongated profile towards the right edge at the junction. At the T-shaped junction, the expansion of DW in a crescent profile extends towards B2 (branch 2) due to the handedness of tilted DW in the input wire. The elongated-DW shows a formation of an asymmetrical semicircle expansion at the T-shaped junction towards B2. The leading edge of the DW in the input wire precede to B2, keeping the tilt angle of the DW with reference to its propagation direction. Shown in Fig. 2(c-③) is the propagation of DW into the other branch 1, labeled as B1 driven by the applications of additional field pulses. From the observation, an input DW configuration in the two-dimensional T-shaped structure changes from a left-handed tilted DW to dendritic-DW, undergoes the bifurcated DW into the branches. Then, DWs within the branches restore the configuration of the identical twin left-handed tilted DWs probably due to the iDMI effective field as it propagates further down through the two branches, B1 and B2. The two DWs in the branches (B1, B2) propagate bidirectionally in their ways, where the DW1 has the clock-wise DW surface rotation and the DW2 propagates without the DW surface rotation, as shown in Fig. 2(c-②~④). The DW1 in B1 shows a propagation with the DW surface orientation changing from left-handed (input wire) to crescent-DW profile (junction), and finally restore the left-handed tilting DW profile (B1, clock-wise rotation), which is identical to the DW configuration in the input wire. Coincidently, the DW2 in B2 keeps moving without changing its initial chirality of DW as in the input wire with little fluctuation of DW configuration. The DW surface rotation and tilt angle change at the junction plays a decisive role to determine which one of the DW1 and DW2 approaches first to the destination pads at the end of the B1 and B2 branches in Fig. 2(c-⑥,⑦) with distinguished DW speed. In the present experiments, the DW2 in B2 reaches the destination first due to the elongation and dendritic-DW profile towards B2 at the junction. Therefore, T-shaped junction also presents an application potential for the sorting of DWs based on their different tilting motion. With further manipulation of DWs in the branches by adjusting the timing and location of DW dynamics in combination with intrinsic materials parameters such as iDMI as well as the local geometry adjustment, application of DW motion for the complex logic and/or memory devices can be expected.

### Evaluation of iDMI effective field

The presence of iDMI is one of the main factors that causes the DW surface to a tilt in the wire and to induce the dendritic-DW motion at the bifurcated junction by perpendicular field application in the present work. The iDMI has been directly measured by a few different methods including Brillouin Light Scattering (BLS) method. Although BLS method is known to have many advantages for the measurement of iDMI, it is noted that the iDMI cannot be measured for our sample by BLS technique. BLS technique has limited frequency resolutions and is difficult to be employed for the measurement in a local area with μm scale dimensions^35,36^. With the sample structure and symmetry used in the present work, the iDMI of our sample is expected to be very small to prevent an irregularly curled DW surface profile instead of a line DW surface profile in the wire. Therefore, the asymmetric circular domain expansion method was utilized, which directly quantifies the effective field induced by the iDMI. The iDMI strength in our film stack was measured by field-induced circular domain expansion method (see the Supplementary Materials for the measurement procedure).

The in-plane field dependence of the DW energy can be expressed with the critical field condition, $|{H}_{x}+{H}_{DMI}| > 4{K}_{D}/\pi \Delta {\mu }_{0}{M}_{s}\equiv {H}_{Neel-Bloch}$, for the transformation of DW type from Bloch DW to Néel DW (the DW energy density is presented in Supplementary Eq. S1). The Néel DW would be favored as the *H*_*DMI*_ value is larger than *H*_*Neel–Bloch*_ in the film. For the case of our film, the critical field condition for the transformation shows $|{H}_{x}+{H}_{DMI}|(240\,{\rm{Oe}}) < {H}_{Neel-Bloch}(830\,{\rm{Oe}})$ (see the Supplementary Materials). For our experiment, the critical separating field to the Néel DW from the Bloch DW is much lower than the critical separating field, +830 Oe in calculation. Therefore, the calculation indicates that the Bloch DW is favored in our experimental conditions. The progressive transition from Bloch to Néel DW according to the iDMI value could be based on a critical iDMI value above *D*_*c*_ ≈ −0.13 mJ/m^2^ based on the theoretical calculation by A. Thiaville *et al*.^37,38^. From the result of asymmetrical circular domain expansion, the measured effective iDMI constant for our film stack is obtained to be *D* ≈ −0.1 mJ/m^2^ (the calculation is shown in the Supplementary Material). In our film, the value of *D* ≈ −0.1 mJ/m^2^ is close to the critical iDMI value, and the sample remains the tilting up-down DW state in the wire.

### Handedness dependence for tilted DW motion

The left-handed or right-handed tilting of DW is induced successfully in the input wire by creation of DW depending on the two opposite directions of initial magnetization. The motions of down-up and up-down DWs depending on the initial magnetization states have been demonstrated. Conversely, the handedness of tilt angle for the down-up DW (right-hand) is opposite to the up-down DW (left-hand) in the input wire, and they can be driven DW with opposite perpendicular field polarities, e.g. a down-up DW (up-down DW) can move by applying −*H*_*z*_ (+*H*_*z*_) field in the illustration of input wire in Fig. 3(a,b). From Kerr microscope images, the tilt angles of DWs in the input wire are determined to be left-handed and right-handed corresponding to the chirality of DWs such as down-up and up-down states of DWs in the input, as shown in Fig. 3(c,d-Input). We note that the motion of DW by pulsed or continuous field in the structure are almost identical to the tilting DW motion in the wire. For a comparison of tilting DWs motion with down-up and up-down magnetization states, asymmetrical expansions of the elongated-DW at the junction towards opposite output branches are observed, as indicated in Fig. 3(c,d-Output ①) with white and black arrows. The iDMI-induced asymmetrical expansion in the elongated-DWs profile at the junction is observed where the right-handed tilting down-up DW is heading to B1 while the left-handed tilting up-down DW is heading to B2. At each branch, the DWs propagate as the spins in the DW surface rotates and restore the same tilt angle as in the input wire in Fig. 3(c,d-Output ②–④). For the B1, down-up and up-down DW1 (DWs in B1) restores the left-handed tilt angle with clock-wise DW surface rotation, as shown in Fig. 3(c,d-Output ②–⑤). Otherwise, the DW2 in B2 keeps the left-handed surface tilt orientation without the significant DW surface rotation. For the movement of DW in the branches, the DW profile may be unstable with some fluctuation caused by the DW pinning from the wire edge roughness during the propagation, as shown in Fig. 3(c,d-Output ③~④) (see the Supplementary Movies 1 and 2). X. Zhang *et al*. reported that the DW surface tension with regard to the iDMI influenced the static and dynamic DW behavior^39^. The DW surface tension can also be considerable to affect the asymmetrical semi-circular DW expansion at the junction. We suppose to consider the Bloch DW which does not show preferred chirality. However, ‘Bloch’-like tilting DWs at the junction have observed the chiral spins inside up-down and down-up DWs with respect to their propagation directions in the micromagnetic simulation. Lastly, the first approach among DWs (DW1 and DW2) from the junction to destination pads clearly shows the strong dependence of the input DWs tilt angle, as shown in Fig. 3(c,d-⑥). The right-handed tilting down-up DW reaches first to the destination pad1. In a similar way, the left-handed tilting up-down DW reaches the destination pad2 prior to the destination pad1.

### Micromagnetic simulation

To understand the influence of iDMI on the observed phenomenon, the tilted DW in the T-shaped structure was investigated by using MuMax^3^ micromagnetic simulation^40^. The structure was discretized with a cell size of 2 nm × 2 nm × 2 nm, and 5μm wire width. The material parameters are chosen to be saturation magnetization *M*_s_ = 750×10^5^ A/m; exchange stiffness *A* = 13pJ/m; damping parameter *α* = 0.1; anisotropy constant,*K*_*perp*_ = 0.6×10^6^ J/m^3^. For a field-driven DW dynamics in the simulation, the DW motion is determined by the expansion/shrinking of domain with respect to the applied out-of-plane field strength. To ensure that a tilted DW is observed in the simulation, an iDMI constant of *D* = −1.2 mJ/m^2^ is assumed. By selecting *D* = −1.2 mJ/m^2^, the left-handed tilting up-down DW can be clearly produced and asymmetrical DW propagation is observed at the junction. For the case of *D* ≲ −0.8 mJ/m^2^, DW has the symmetrical motion without any tilt angle of DW profile in the structure. For *D* ≥ −2.5 mJ/m^2^, the stronger iDMI causes a DW distortion and elongated stripe domain in the wire. Moreover, a selective propagation between the branches is no longer present. The value of iDMI was measured *D* ≈ −0.1 mJ/m^2^ for our sample in the experiment, but simulation result is matched better to the experiment result with *D* ≈ −0.2 mJ/m^2^ (see Supplementary Movies 1 and 2). First of all, the time scale of the experiments is in the order of ~0.1 s, while the time scale of the simulation is limited to ~10 ns. And the DW velocity in experiments is in the order of ~100 μm/s, while it is approximately 400 m/s in micromagnetic simulations in order to move the DW in the ~10 μm scale wire within the proper time scale. Large iDMI value is able to induce the tilted DW adequately and prevents the Walker breakdown at high field. Since the DW tilt angle is a function of many parameters including not only the iDMI but also *H*_*z*_, it is difficult to find the exact relationship between DW tilt angle and the parameters. Hence, the relation was checked using one-dimensional DW dynamics simulator^41^, and it can be found that small iDMI with high field strength is difficult to induce a tilt angle for the DW surface (See the Supplementary Materials).

**Figure 4:**
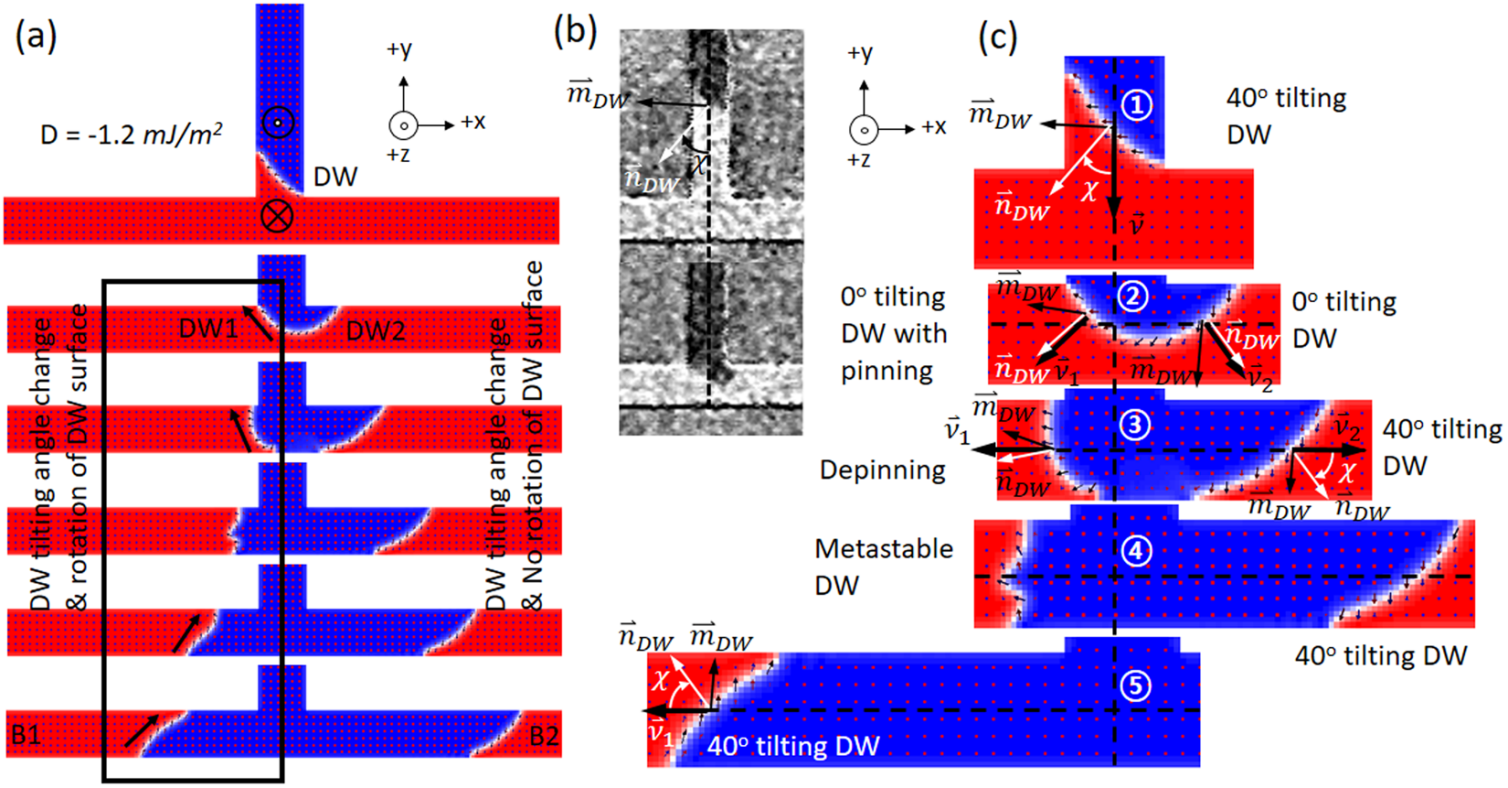
(**a**) Micromagnetic simulation results representing the evolution of the DW position and configuration for a T-shaped device, which has a wire width, 5 μm. An up-down DW configuration has been tested in simulation. (**b**) Differential Kerr microscopy images for DW tilt angle (χ) in the input wire and at the bifurcated junction. (**c**) DW tilt angle has been oriented due to the propagation directions from an input wire to two output branches.

Figure 4(a) shows that a left-handed titling up-down DW propagates and tilt angle of DW1 surface rotates in clock-wise orientation in black box via the T-shaped structure. Shown in Fig. 4(b,c) compares the tilting DW profiles from the Kerr image and simulated DWs as a DW propagates via T-shaped structure. The Kerr images and simulation results shows high resemblance with ~ 40° tilting of DW surface with respect to the DW propagation direction in the input wire. The tilted DW propagation induces the asymmetric expansion at the bifurcated junction with a crescent profile in Fig. 4(c-②). The 40° tilt angle cannot be sustained for the case of a crescent and metastable DW profile shown in Fig. 4(c-②~④) around the junction.

The internal spin dynamics of DW configuration at the junction depicts on the simulation results in Fig. 4(c). The normal vector of the DW surface, internal spin orientation of DW, DW tilt angle, and propagation direction of DWs in T-shaped structure describes ${\overrightarrow{n}}_{DW},\,{\overrightarrow{m}}_{DW},\,\chi ,\,\nu ,\,{\nu }_{1},\,{\nu }_{2}$, respectively, in Fig. 4(c). For the initial position of the DW in the input wire, a DW tilt angle is set to be a χ ≈ 0° in the simulation. The external field + *H*_*z*_ applied along + *z*-axis (up) will expand the + *M*_*z*,_ components within the wire. As a DW propagates via input wire, the iDMI subsequently stabilizes a χ ≈ 40° tilting DW profile with an up-down DW configuration having an internal spins, ${\overrightarrow{m}}_{DW}$, pointing along the -x-axis in Fig. 4(c-①). As such, the field induced an up-down ‘Bloch’-like DW in the input wire will move along *−y*-direction in 1-dimensional input wire system. At the junction, the internal spins orientation within the DW1 are aligned parallel to the propagation direction as the DW(input) passes via the right corner at the junction in Fig. 4(c-②,③). The crescent profile of DW at the junction indicates that there is no tilt angle (χ ≈ 0°) of the DW profile with respect to the direction of expansion in Fig. 4(c-②). The DW expands asymmetrically in both branches as the field strength increases along +*H*_*z*_. Figure 4(c) indicates that the DW tilt angle rotates from χ ≈ 40° to χ ≈ 0° and restore again to χ ≈ 40° as a tilting DW passes via the junction along the B1, as shown in Fig. 4(c-①~⑤). Note that the metastable DW profile with respect to the propagation direction observes only at the B1. The DW2 in B2 adequately keeps the left-handed tilted profile without any torsion of DW profile. The DW configuration has a dynamical change as a DW propagates towards B1 from the junction, which means DWs in branches restore a symmetric handedness of DWs profile comparably in B1 and B2, as shown in Fig. 4(c-④,⑤). Besides, the pinning of DW1 at the junction induces that the DW1 surface and the internal DW spin rotates 90° in clock-wise orientation as comparing the ${\overrightarrow{n}}_{DW},\,{\overrightarrow{m}}_{DW}$ angles in between Fig. 4(c-①,⑤). The process of internal DW spin rotation drives an asymmetrical DW spreading at the junction to recover a symmetric propagation of DWs in terms of DWs surface rotation in the branches, as shown in Fig. 4(c). The DW1 surface orientation modulation and asymmetrical DWs propagation at the junction can be caused by the iDMI effective field and structural corners in the T-shaped junction.

The changes of DW speed has previously been reported when the DW surface has a tilt angle with respect to its propagation direction^25^. However, the tilted DWs position and profile transformation are able to modulate or modify the speed of DW with an asymmetrical propagation at the junction structure due to the iDMI effect. Additionally, the pinning process of internal DW spin at the corners of junction can assist a DW to restore its original profile in the structure. In our micromagnetic simulation, the existence of the asymmetrical DW expansion and motions in the T-shaped structure was essentially reproduced in consistency with the experimental observations with Kerr microscopy.

## Conclusion

In conclusion, we investigate the asymmetrical propagation of DW along the T-shaped paths. DW bifurcates at the T junction and the divided DWs turn around the junction in series whose order is determined by the chirality of the spin structures at the front of the DW. Therefore, one of the DWs reaches earlier than the other to the destination pads at the ends of the branches. The DW undergoes a structural re-orientation for the tilt angle in branches to induce a different process of reaching to the destination pads by a uniform external field. The structural effect at the T-shaped junction corner and iDMI-induced DW tilt angle rotation make DWs to move at different speeds. We show that selective tilted DW motion and controlling the handedness of tilt orientation of DW in T-shaped structure can be achieved by utilizing iDMI and geometry. It is noted that the magnitude of the iDMI effect should be considered in the design of magnetic DW track circuit or the network of paths for DW propagation. Conversely, information on the iDMI can be extracted by measuring the difference of the DW propagation speed through the T-junction.

## Methods

### Sample preparation

A 200-nm-thick SiO_2_ covered silicon wafer has been used as a substrate for Co/Pt multilayer deposition using ultra-high vacuum DC magnetron sputtering system at room temperature. The substrates were cleaned with acetone and Iso-propyl alcohol for each 20 mins under ultrasonic agitation, and quickly blow dried with N_2_ gas. Then, the device was fabricated in sequence; T-shaped structure was patterned with positive photo-resist by photo-lithography. The sample etching were completed using Ar + ion milling technique. The photo-resist was removed by acetone, followed by cleaning process for each 10 mins.

### Kerr microscope

A home-made perpendicular Kerr microscope was employed to capture the presence of DWs motion in the sample. The magnetized sample with the presence of DW was exposed to an incident linear polarized light. The green LED was used as the incident light source. The reflected light from the magnetized sample was monitored by the analyzer, which is adjacently located cross polarizer. The reflected light was recorded by a CCD camera. The final Kerr images was obtained by the subtraction of two images; First is for the image of magnetically saturated sample and second is for the image of present DW in the sample. The image subtraction was processed in Matlab to obtain the final Kerr images.

## Electronic supplementary material

Supplementary Information

Supplementary Movie1

Supplementary Movie2

## References

[1] Allwood DA (2005). Magnetic Domain-Wall Logic. Science.

[2] Allwood DA (2002). Submicrometer Ferromagnetic NOT Gate and Shift Register. Science.

[3] Parkin SS, Yang SH (2015). Memory on the racetrack. Nat. Nanotech..

[4] Kobs A (2011). Anisotropic Interface Magnetoresistance in Pt/Co/Pt Sandwiches. Phys. Rev. Lett..

[5] Ryu KS, Thomas L, Yang SH, Parkin SS (2013). Chiral spin torque at magnetic domain walls. Nat. Nanotech..

[6] Brataas A (2013). Chiral domain walls move faster. Nat. Nanotech..

[7] Woo S (2014). Enhanced spin-orbit torques in Pt/Co/Ta heterostructures. Appl. Phys. Lett..

[8] Haazen PP (2013). Domain wall depinning governed by the spin Hall effect. Nat. Mater..

[9] Dzyaloshinskii IE (1957). Thermodynamic Theory of “Weak” Ferromagnetism In Antiferromagnetic Substances. Sovient Physics JETP.

[10] Moriya T (1960). Anisotropic Superexchange Interaction and Weak Ferromagnetism. Phys. Rev..

[11] Fert A, Cros V, Sampaio J (2013). Skyrmions on the track. Nat. Nanotech..

[12] Cho J (2017). The sign of the interfacial Dzyaloshinskii–Moriya interaction in ultrathin amorphous and polycrystalline magnetic films. J. Phys. D: Appl. Phys..

[13] Emori S (2014). Spin Hall torque magnetometry of Dzyaloshinskii domain walls. Phys. Rev. B.

[14] Khvalkovskiy AV (2013). Matching domain-wall configuration and spin-orbit torques for efficient domain-wall motion. Phys. Rev. B.

[15] Pai C-F (2012). Spin transfer torque devices utilizing the giant spin Hall effect of tungsten. Appl. Phys. Lett..

[16] Garg C, Yang SH, Phung T, Pushp A, Parkin SS (2017). Dramatic influence of curvature of nanowire on chiral domain wall velocity. Sci. Adv..

[17] Teoh HK, Goolaup S, Lew WS (2017). Dzyaloshinskii–Moriya interaction induced domain wall depinning anomaly in ferromagnetic nanowire. J. Phys. D: Appl. Phys..

[18] Tchernyshyov O, Chern G-W (2005). Fractional Vortices and Composite Domain Walls in Flat Nanomagnets. Phys. Rev. Lett..

[19] Kim KJ (2013). Two-barrier stability that allows low-power operation in current-induced domain-wall motion. Nat. Commun..

[20] Franken JH, Hoeijmakers M, Lavrijsen R, Swagten HJ (2012). Domain-wall pinning by local control of anisotropy in Pt/Co/Pt strips. J. Phys. Condens. matter.

[21] Miron IM (2011). Fast current-induced domain-wall motion controlled by the Rashba effect. Nat. Mater..

[22] Boulle O (2013). Domain Wall Tilting in the Presence of the Dzyaloshinskii-Moriya Interaction in Out-of-Plane Magnetized Magnetic Nanotracks. Phys. Rev. Lett..

[23] Heide M, Bihlmayer G, Blügel S (2008). Dzyaloshinskii-Moriya interaction accounting for the orientation of magnetic domains in ultrathin films: Fe/W(110). Phys. Rev. B.

[24] Je S-G (2013). Asymmetric magnetic domain-wall motion by the Dzyaloshinskii-Moriya interaction. Phys. Rev. B.

[25] Hrabec A (2014). Measuring and tailoring the Dzyaloshinskii-Moriya interaction in perpendicularly magnetized thin films. Phys. Rev. B.

[26] Ryu K-S, Thomas L, Yang S-H, Parkin SS (2012). Current Induced Tilting of Domain Walls in High Velocity Motion along Perpendicularly Magnetized Micron-Sized Co/Ni/Co Racetracks. Appl. Phys. Express.

[27] Choi YH (2016). Field-driven domain wall motion under a bias current in the creep and flow regimes in Pt/[CoSiB/Pt]_N_ nanowires. Sci. Rep..

[28] Martinez E, Emori S, Perez N, Torres L, Beach GSD (2014). Current-driven dynamics of Dzyaloshinskii domain walls in the presence of in-plane fields: Full micromagnetic and one-dimensional analysis. J. Appl. Phys..

[29] Jué E (2016). Chiral damping of magnetic domain walls. Nat. Mater..

[30] Yang H, Boulle O, Cros V, Fert A, Chshiev M (2018). Controlling Dzyaloshinskii-Moriya Interaction via Chirality Dependent Atomic-Layer Stacking, Insulator Capping and Electric Field. Sci. Rep..

[31] Soumyanarayanan A (2017). Tunable room-temperature magnetic skyrmions in Ir/Fe/Co/Pt multilayers. Nat. Mater..

[32] Yang H, Thiaville A, Rohart S, Fert A, Chshiev M (2015). Anatomy of Dzyaloshinskii-Moriya Interaction at Co/Pt Interfaces. Phys. Rev. Lett..

[33] Koyama T (2012). Current-induced magnetic domain wall motion below intrinsic threshold triggered by Walker breakdown. Nat. Nanotech..

[34] Koyama T (2011). Observation of the intrinsic pinning of a magnetic domain wall in a ferromagnetic nanowire. Nat. Mater..

[35] Cho J (2015). Thickness dependence of the interfacial Dzyaloshinskii–Moriya interaction in inversion symmetry broken systems. Nat. Commun..

[36] Kim N-H (2016). Interfacial Dzyaloshinskii-Moriya interaction, surface anisotropy energy, and spin pumping at spin orbit coupled Ir/Co interface. Appl. Phys. Lett..

[37] Tetienne JP (2015). The nature of domain walls in ultrathin ferromagnets revealed by scanning nanomagnetometry. Nat. Commun..

[38] Thiaville A, Rohart S, Jué É, Cros V, Fert A (2012). Dynamics of Dzyaloshinskii domain walls in ultrathin magnetic film. EPL (Europhysics Letters).

[39] Zhang X (2018). Direct Observation of Domain-Wall Surface Tension by Deflating or Inflating a Magnetic Bubble. Phys. Rev. Applied.

[40] Vansteenkiste A (2014). The design and verification ofmumax3. AIP Adv..

[41] Kim H, Heo SW, You C-Y (2017). Implementation of one-dimensional domain wall dynamics simulator. AIP Advances.

