# Supplementary material for: Bidirectional propagation of tilting domain walls in perpendicularly magnetized T shaped structure with the interfacial Dzyaloshinskii-Moriya interaction
Source: Sci Rep. 2018 Dec 21;8:18035. doi: 10.1038/s41598-018-36523-9 (PMC6303305; doi:10.1038/s41598-018-36523-9)
Supplement: Supplementary file 1 — Supplementary Information [file 41598_2018_36523_MOESM1_ESM.docx]

**SUPPLEMENTARY MATERIALS**

**Bidirectional propagation of tilting domain walls in perpendicularly magnetized T shaped structure with the Dzyaloshinskii Moriya interaction**

Jaesuk. Kwon, Hee-Kyeong Hwang, Jung-Il Hong, and Chun-Yeol You

Department of Emerging Material Science, DGIST, Daegu 42988, South Korea

**Table of contents**

**S1. Evaluation of iDMI effective field.**

**S2. DW energy density for the transition.**

**S3. Tilt angle variation in 1-dimensional simulator**

**S1. Evaluation of iDMI effective field.**

For the purpose of characterizing the iDMI strength, iDMI effective field is measured by field induced asymmetrical circular domain expansion method.^[[1]](#endnote-1)^ Fig. S1 (a) shows that an asymmetric domain expansion caused by applying a perpendicular field combined with an in-plane field. The asymmetric domain expansion is observed by applying an additional in-plane field with symmetric expansion of domain by a fixed perpendicular field. A circular domain (up-down DW) has experiences of asymmetric expansion related to the velocity increase along the applied $\pm H_{x}$, as following the effective magnetic field $H_{x}+H_{DMI}$ in Fig. S1 (b) and (c).


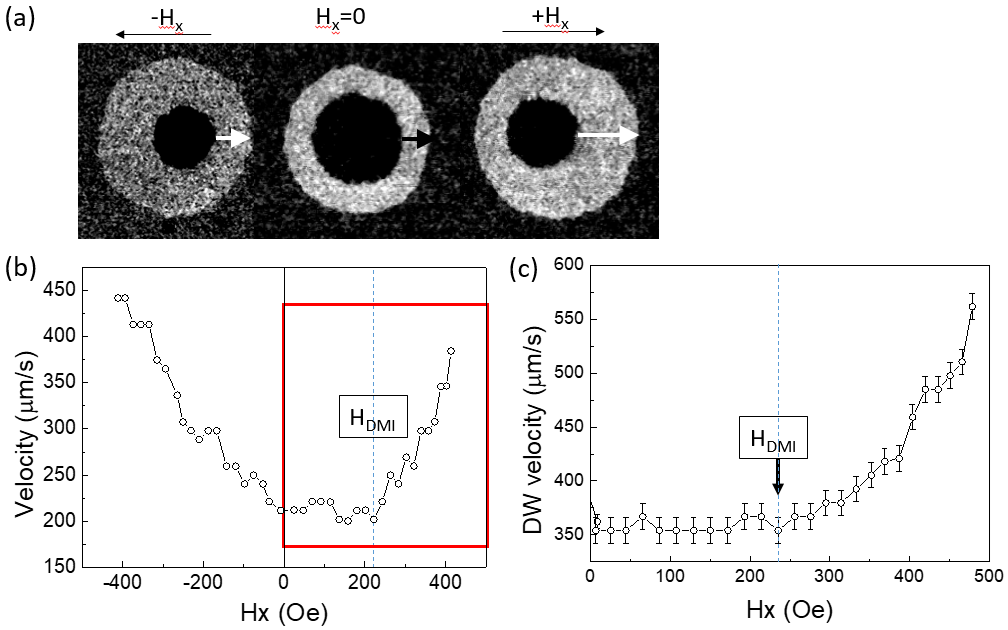


**Figure S1.** (a) Circular domain expansion induced by perpendicular field. An asymmetrical expansion drives with applying in-plane field, *±H_x_*. The images are captured as field pulses-driven circular domain expansion with pulse duration of 100 msec. (b) The asymmetry of velocity in circular domain expansion as function of *±H_x_*. (c) The $H_{DMI}$ value observed at +240 Oe as the asymmetric expansion shows in circular domain by in-plane file.

The obtained value of iDMI effective field, $H_{DMI}$, on Pt/[Co/Pt]_x4_/Ta multilayered film was estimated ~ +240 Oe along the +x-axis, as shown in Fig. S1 (c) with large uncertainty due to the broad minimum. From the result of asymmetrical circular domain expansion, the measured effective iDMI constant for our film stack is obtained ${D=\mu}_{0}H_{DMI}M_{s}\Delta\approx-0.1 mJ/m^{2}$. The measured parameters for our thin film are; $M_{s} =1\times{10}^{6}A/m$, $H_{k} =1T$,$K_{0} =\mu_{0}(H_{K}M_{s}-M_{s}^{2})/2=6.2\times{10}^{5} J/m^{3}$. The DW width was estimated as $\Delta=\sqrt{A/K_{0}}=5\mathrm{nm}$, where the exchange constant is $A =16 pJ/m$.^[[2]](#endnote-2)^ In our film, the value of $D\approx-0.1 mJ/m^{2}$ is smaller than the critical value the progressive transformation of DW from Bloch DW to Néel DW,^[[3]](#endnote-3)^ but the sample remains the tilting up-down DW state in the wire.

**S2. DW energy density for the transition.**

The in-plane field dependence of the DW energy ^[[4]](#endnote-4)^ can be expressed as

$\sigma\left( H_{x} \right)=\left\{ \begin{aligned} \sigma_{0}-\frac{\pi^{2}\Delta\mu_{0}M_{s}^{2}}{8K_{D}}\left( H_{x}+H_{DMI} \right)^{2} for \left| H_{x}+H_{DMI} \right|<\frac{4K_{D}}{\pi\Delta\mu_{0}M_{s}} \\ \sigma_{0}+2K_{D}\Delta-\pi\Delta\mu_{0}M_{s}\left| H_{x}+H_{DMI} \right| otherwise \end{aligned} \right.$, (S1)

where $M_{s}$ is saturation magnetization, $\sigma_{0}$ Bloch wall energy density, $\Delta$ the DW width, $K_{D}$ the DW anisotropy energy. In the case of DW energy condition as the Bloch DW is fully transformed into a Néel DW, the condition is given by $\left| H_{x}+H_{DMI} \right|>4K_{D}/\pi\Delta\mu_{0}M_{s}\equiv\mu_{0}H_{Neel-Bloch}$, where the $M_{s}$ is saturation magnetization$M_{s} =1\times{10}^{6}A/m$, the DW width $\Delta=5\mathrm{nm}$, and the DW anisotropy energy $K_{D} =N_{x}\mu_{0}M_{s}^{2}/2=6.5\times{10}^{4}J/m^{3}$ where the $N_{x}$ is the demagnetizing coefficient of the DW.

The critical field separating the Néel DW from the Bloch DW was calculated to be $\mu_{0} H_{Neel-Bloch}=830 \mathrm{Oe}$ in our film stack. From the boundary condition of DW transformation, the calculation shows$\left| H_{x}+H_{DMI} \right|(240 \mathrm{Oe})<\mu_{0}H_{Neel-Bloch}(830 \mathrm{Oe})$.

**S3. Tilt angle variation in 1-dimensional simulator**

The tilt angle of DW in one-dimensional system such as straight wire could be settled by the iDMI value in this study. The standalone one-dimensional DW motion simulator (1-D simulator) by Kim *et al*.^[[5]](#endnote-5)^ has been used for the investigation of time-dependent tilt angle changes influenced by various iDMI values and external fields. The parameters used for 1-D simulator are a saturation magnetization $M_{s} =0.7\times{10}^{6}A/m$, an uniaxial anisotropy energy $K_{U}=4.8\times{10}^{5} J/m^{3}$, an damping constant $\alpha=0.1$, an exchange stiffness constant $A_{ex} =13 pJ/m$, and a wire width (y-axis) $L_{y}=5 \mu m$, and thickness (y-axis) $L_{z}=4 \mathrm{nm}$.

The initial DW configuration has been chosen a Bloch type with absence of external fields. The internal DW magnetization angle for Bloch type in simulator is adopted $\Psi=\pi/2$. External perpendicular field $B_{Z}=30 \mathrm{mT}$ was applied for driving an up-down DW in the wire by support a tilt angle of DW with $B_{y}=-0.25 \mathrm{mT}$. Those field values for DW tilt angle are implemented from the report by Martinez *et al*. ^[[6]](#endnote-6)^ The iDMI constant $D=-1.2 mJ/m^{2}$ used in 1-D simulator and MuMax^3^ micromagnetic simulation is a value to induce the tilt angle of DW $\chi\approx{40}^{o}$. Concurrently, the DW has been kept the tilt angle $\chi\approx{40}^{o}$ during propagation in the wire. The angle$\chi\approx{40}^{o}$ is almost matched with a DW profile in the experiment, 1D simulator, and MuMax^3^ micromagnetic simulation results.


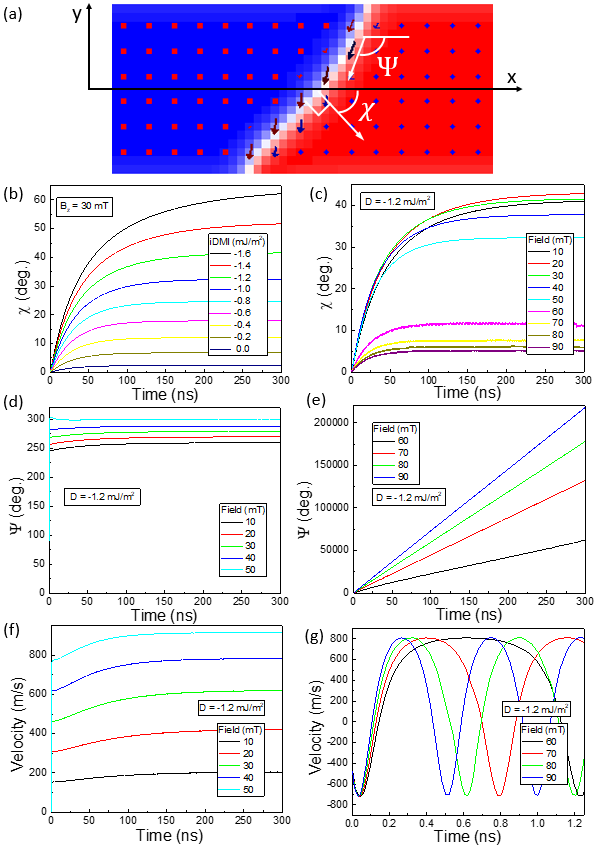


**Figure S2.** One-dimensional domain wall (DW) dynamics simulator results. (a) Micromagnetic snapshot of left-handed tilting DW in motion. $\Psi$ represents the angle of internal DW magnetization, and $\chi$ is the tilt angle of the DW. (b) The evolution of DW angle due to various iDMI values in field-driven DW dynamics by applying, *±H_z_*. (c) The tilt angle of DW is influenced by perpendicular field strength. (d) The internal DW magnetization angle change in the range of perpendicular field from 10 mT to 50 mT, and (e) the range of field from 60 mT to 90 mT. (f) DW velocity linearly increases as function of external field. (g) Oscillating of velocity for a DW indicates that the average speed of DW decreases as the external field increases more than 60 mT.

The total running time of simulator is 300 ns when the tilt angle is stabilized as shown in Fig. S2. From the results, we notice that the tilt angle is linearly increased due to the increment of iDMI value as shown in Fig. S2 (b). The angle is set to be 40^o^ in the value of $D=-1.2 mJ/m^{2}$ with the magnetic parameters in the 1D simulator. The tilt angle strongly dominates by the iDMI value, and the speed of DW relies on the perpendicular field strength. The speed of DW in the 1D simulator shows $v\approx500 m/s$ as the external perpendicular field 30mT applied. The DW speed is also slightly affected to increases with the increment of the iDMI value. For the case of perpendicular field strength, the tilt angle is abruptly reduced above 60 mT shown in Fig. S2 (c). It is well known that the internal DW magnetization angle $\Psi$ is constant before Walker breakdown occurs, as shown in Fig. S2 (d). For Fig. S2 (e), the internal DW magnetization angle varies continuously after the Walker breakdown occurs at the field value above 60 mT, which is the critical field value of tilt angle of DW, $\chi\approx{40}^{o}$. The 1-D simulator result indicates that the small iDMI ($D\ll-1.2 mJ/m^{2}$) with high field strength ($B_{Z}\gg50 \mathrm{mT}$) is difficult to induce a tilt angle of DW in the wire. In addition to, the velocity of DW linearly increases as the perpendicular field increases up to 50 mT in Fig. S2 (f), then oscillating velocity alternating its sign obtained with applying external field above 60 mT in Fig S2 (g). We note that the angle $\Psi$ is continuously rotating (increasing in Fig. S2 (e)) while DW position is move forward and backward with different amplitudes, means that the DW is slowly moving forwards in a large time scale with oscillating in the Walker breakdown ^5^.

**References**

1. Je, S.-G. *et al.* Asymmetric magnetic domain-wall motion by the Dzyaloshinskii-Moriya interaction. *Phys. Rev. B* **88**, 214401, doi: 10.1103/PhysRevB.88.214401 (2013). [↑](#endnote-ref-1)
2. Boulle, O. *et al.* Domain Wall Tilting in the Presence of the Dzyaloshinskii-Moriya Interaction in Out-of-Plane Magnetized Magnetic Nanotracks. *Phys. Rev. Lett.* **111**, 217203, doi: 10.1103/PhysRevLett.111.217203 (2013). [↑](#endnote-ref-2)
3. Tetienne, J. P. *et al*. The nature of domain walls in ultrathin ferromagnets revealed by scanning nanomagnetometry. *Nat. Commun*. **6**, 6733, doi: 10.1038/ncomms7733 (2015). [↑](#endnote-ref-3)
4. Thiaville, A. Rohart, S. Jué, É. Cros, V. & Fert, A. Dynamics of Dzyaloshinskii domain walls in ultrathin magnetic film. *EPL (Europhysics Letters)* **100**, 57002, doi: 10.1209/0295-5075/100/57002 (2012). [↑](#endnote-ref-4)
5. Kim, H. Heo, S. W. & You, C.-Y. Implementation of one-dimensional domain wall dynamics simulator. *AIP Advances* **7**, 125231, doi: 10.1063/1.4996029 (2017). [↑](#endnote-ref-5)
6. E. Martinez, *et al*, Current-driven dynamics of Dzyaloshinskii domain walls in the presence of in-plane fields: Full micromagnetic and one-dimensional analysis. *J. of Appl. Phys*. **115**, 213909, doi: 10.1063/1.4881778 (2014). [↑](#endnote-ref-6)
